# Phage-Like *Streptococcus pyogenes* Chromosomal Islands (SpyCI) and Mutator Phenotypes: Control by Growth State and Rescue by a SpyCI-Encoded Promoter

**DOI:** 10.3389/fmicb.2012.00317

**Published:** 2012-08-30

**Authors:** Julie Scott, Scott V. Nguyen, Catherine J. King, Christina Hendrickson, W. Michael McShan

**Affiliations:** ^1^Department of Pharmaceutical Sciences, The University of Oklahoma Health Sciences CenterOklahoma City, OK, USA; ^2^Department of Microbiology and Immunology, The University of Oklahoma Health Sciences CenterOklahoma City, OK, USA

**Keywords:** prophages, chromosomal islands, SpyCI, DNA mismatch repair, mutator phenotype, group A streptococcus, *Streptococcus pyogenes*

## Abstract

We recently showed that a prophage-like *Streptococcus pyogenes* chromosomal island (SpyCI) controls DNA mismatch repair and other repair functions in M1 genome strain SF370 by dynamic excision and reintegration into the 5′ end of *mutL* in response to growth, causing the cell to alternate between a wild type and mutator phenotype. Nine of the 16 completed *S. pyogenes* genomes contain related SpyCI integrated into the identical attachment site in *mutL*, and in this study we examined a number of these strains to determine whether they also had a mutator phenotype as in SF370. With the exception of M5 genome strain Manfredo, all demonstrated a mutator phenotype as compared to SpyCI-free strain NZ131. The integrase gene (*int*) in the SpyCIM5 contains a deletion that rendered it inactive, and this deletion predicts that Manfredo would have a pronounced mutator phenotype. Remarkably, this was found not to be the case, but rather a cryptic promoter within the *int* ORF was identified that ensured constitutive expression of *mutL* and the downstream genes encoded on the same mRNA, providing a striking example of rescue of gene function following decay of a mobile genetic element. The frequent occurrence of SpyCI in the group A streptococci may facilitate bacterial survival by conferring an inducible mutator phenotype that promotes adaptation in the face of environmental challenges or host immunity.

## Introduction

Bacteria and bacteriophages exist in a dynamic relationship. In *Streptococcus pyogenes* (group A streptococcus), prophages are prominent components of bacterial chromosomes (Banks et al., 2002; Canchaya et al., 2002). The integration of phage DNA into a bacterial chromosome imparts an increased metabolic burden on the host and serves as a constant threat to survival should conditions change and the prophage enter the lytic cycle. However, the prevalence of lysogeny in *S. pyogenes* implies that prophage acquisition benefits the host under some conditions. Phage-encoded fitness factors such as toxin genes act as means of bringing positive selection pressure upon the host bacterium to maintain the phage DNA in its chromosome, and while such fitness factors may not be required for the phage life cycle, they may act to promote host bacterial growth or survival (Brussow et al., 2004).

**Figure 1:**
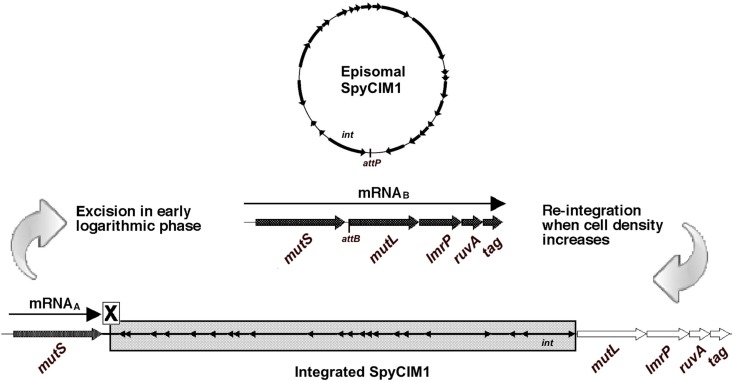
**MMR regulation by SpyCIM1 in strain SF370**. Prophage-like chromosomal island SpyCIM1 integration separates *mutL*, *lmrP*, *ruvA*, and tag from the promoter upstream of *mutS*, preventing expression of these genes and leading to a mutator phenotype in strain SF370 (Scott et al., 2008). *mutS* is constitutively expressed from mRNA_A_, which is truncated by the presence of the SpyCI. When cells enter early logarithmic growth phase, approximately at the time of initiation of DNA replication, the SpyCI excises from the host chromosome and restores transcription of the polycistronic message from *mutS* to tag (mRNA_B_). The excised phage circularizes and replicates in the host cell as an episome. As cell densities increase, the phage genome integrates into *attB* located at the 5′ end of *mutL*, returning the cell to the mutator phenotype. Large, unfilled arrows indicate transcriptionally inactive genes of the MMR operon (*mutS*, *mutL*, *lmrP*, *ruvA*, and tag), while transcriptionally active ones are large arrows filled in black. Small, filled arrows indicate the SpyCI ORFs; except for *int* (Scott et al., 2008), the transcriptional patterns of the SpyCI genes are unknown at this time.

We recently reported a novel prophage-like chromosomal island (CI) in M1 genome strain SF370 that integrates and excises from the chromosome in response to cell growth stage (Scott et al., 2008). This element, originally annotated as prophage SF370.4, is now identified as *S*. *pyogenes* Chromosomal Island M1 (SpyCIM1) to conform to the nomenclature proposed by Novick et al. (2010). SpyCIM1 integrates between the DNA mismatch repair (MMR) genes *mutS* and *mutL*, leading to the silencing of *mutL* and several downstream genes, resulting in the inactivation of MMR. However, during exponential growth the SpyCI excises and replicates as an episome, allowing *mutL* to be transcribed, and restoring MMR to correct errors following DNA replication (Figure 1). As cell density increases, SpyCIM1 re-integrates into the chromosome, interrupting transcription of the polycistronic message and creating a mutator phenotype (Scott et al., 2008). Three genes directly downstream of *mutL* are predicted to be included on this polycistronic message: the multidrug resistance transporter *lmrP*, the Holliday junction DNA helicase *ruvA*, and the base excision repair glycosylase *tag*. The SpyCI are members of a larger group of bacteriophage-related CI found in the low %G + C Gram-positive organisms. The best-characterized members of this group are the prophage-like *Staphylococcus aureus* pathogenicity islands (SaPI), which are the vectors for the toxic shock syndrome toxin and other virulence factors (Novick et al., 2010). The SaPI disseminate to new host staphylococcal cells by hijacking and remodeling the capsids of helper bacteriophages (Tormo et al., 2008; Ubeda et al., 2009), and a similar strategy appears to be used by the SpyCI to infect new host streptococci (Nguyen and McShan, unpublished results). Thus, these CI may be considered a unique subset of prokaryotic viruses.

Nine of the 16 published *S. pyogenes* genomes have a SpyCI integrated between *mutS* and *mutL*. Although integration in this location appears to be a common occurrence in *S. pyogenes*, limited sequence identity has been observed between these modified prophages with the exception of highly conserved modules for site-specific integration, DNA replication, and maintenance (Scott et al., 2008). Here we report that *S. pyogenes* strains with a SpyCI integrated between the MMR genes *mutS* and *mutL* exhibit an increased spontaneous mutation rate and an increased sensitivity to UV irradiation due to the inhibition of RuvA as compared to strains lacking these elements. The unexpected exception was the M5 genome strain Manfredo that is always wild type for mutation rate and UV sensitivity, despite having a CI permanently integrated between *mutS* and *mutL* due to a 128 bp deletion in the integrase ORF. We found that this paradox of harboring a SpyCI without a mutator phenotype is a result of the transcription of *mutL* and the downstream genes from a cryptic promoter within the inactivated SpyCI integrase pseudogene, providing a novel example of host gene expression rescue following CI sequence decay.

## Materials and Methods

### Bacterial strains and culture conditions

Bacterial strains used in this work are listed in Table 1. *S. pyogenes* NZ131 is an M49 strain isolated from a patient in New Zealand with acute post-streptococcal glomerulonephritis; its genome has been completely sequenced and does not have a SpyCI integrated between the MMR genes, *mutS* and *mutL* (McShan et al., 2008). Thus, it is wild type for MMR and has been used previously as a control (Scott et al., 2008). The following strains, whose genomes have been completely sequenced, were available for these studies, and all contain a SpyCI integrated into the identical attachment site in *mutL*: SF370 (M1), MGAS10270 (M2), MGAS10750 (M4), Manfredo (M5), MGAS10394 (M6), and MGAS6180 (M28; Table 1). *S. pyogenes* strains 270fer, 3470-04, and 5474-00 are serotype M49 clinical isolates whose genomes have not been sequenced but have a prophage-like SpyCI integrated at *mutL* (McShan et al., 2008). These SpyCI containing clinical strains were included to provide serotype-matched controls for NZ131. *S. pyogenes* strains were grown in Todd-Hewitt broth (BD, Franklin Lakes, NJ, USA) supplemented with 2% yeast extract (THY medium) at 37°C. For some strains, brain heart infusion (BHI) supplemented with 5% heated horse serum was used for growth media. *Escherichia coli* strains were grown in L broth supplemented with the appropriate antibiotics as needed (Sambrook and Russell, 2001). Growth was monitored by absorbance at 600 nm (*A*_600_) using a Genesys20 spectrophotometer (Thermo Scientific, Waltham, MA, USA), and cells were harvested at appropriate growth stages as needed for subsequent experimental analysis. For induction of SpyCI, cultures were grown until the *A*_600_ = 0.2 and then split into two 40 ml portions. Samples were induced by the addition of mitomycin C from *Streptomyces caespitosus* (Calbiotech, Spring Valley, CA, USA) to a final concentration of 0.2 μg/ml. Incubation at 37°C was continued, and samples were collected for each strain at approximately *A*_600_ = 0.2, 0.4, 0.6, and 0.8 and stored in RNA later (Ambion, Austin, TX, USA) at 4°C. The remainder of each culture was left at 37°C overnight, and final samples were collected for each strain the following day.

**Table 1:** ***Streptococcus pyogenes* strains used in this work**.

Strain	Relevant genotype	Associated disease	Accession number (ref.)
NZ131	*emm49*, MMR wild type	Acute post-streptococcal glomerulonephritis	CP000829 McShan et al. (2008)
SF370	*emm1*, SpyCIM1	Wound infection	AE004092 Ferretti et al. (2001), Scott et al. (2008)
MGAS10270	*emm2*, SpyCIM2	Pharyngitis	CP000260 Beres et al. (2006)
MGAS10750	*emm4*, SpyCIM4	Pharyngitis	CP000262 Beres et al. (2006)
Manfredo	*emm5*, SpyCIM5	Rheumatic heart disease	AM295007 Holden et al. (2007)
MGAS10394	*emm6*, SpyCIM6	Pharyngitis	CP000003 Banks et al. (2004)
MGAS6180	*emm28*, SpyCIM28	Invasive disease	CP000056 Green et al. (2005)
3470-04	*emm49*, SpyCIM49.1	Invasive disease	McShan et al. (2008)
5474-00	*emm49*, SpyCI49.2	Invasive disease	McShan et al. (2008)
270fer	*emm49*, SpyCIM49.3	Invasive disease	McShan et al. (2008)
OKM48	Manfredo (pWM483)		This work
OKM49	Manfredo (pWM483)		This work
OKM50	NZ131 (pWM483)		This work
OKM51	NZ131 (pWM483)		This work
OKM52	NZ131 (pDL276)		This work
OKM54	NZ131 (pWM488)		This work
OKM55	NZ131 (pWM489)		This work
OKM56	NZ131 (pWM490)		This work
OKM57	Manfredo (pWM488)		This work
OKM58	Manfredo (pWM488)		This work
OKM59	Manfredo (pWM490)		This work
OKM60	Manfredo (pWM490)		This work
OKM61	Manfredo (pWM489)		This work

### Nucleic acid isolation and cDNA synthesis

Isolation of streptococcal DNA was done as previously reported (Pitcher et al., 1989; McShan et al., 1998). DNA concentrations were determined by spectroscopy at 260 nm using a NanoDrop ND-1000 spectrophotometer (NanoDrop Products, Wilmington, DE, USA). RNA was prepared using the RiboPure system for bacteria (Ambion) by following the manufacturer’s recommended protocol. RNA samples were tested for DNA contamination by PCR using primers specific for the variable region of the *emm* gene (Beall et al., 1996). RNA samples were converted to cDNA using Superscript II (Invitrogen, Carlsbad, CA, USA) and random hexamer priming by following the manufacturer’s protocol.

### PCR and quantitative real-time PCR (qRT-PCR)

PCR was used to detect transcription of *mutS*, *mutL*, and the intergenic region between *int* and *mutL*. Ten nanograms of cDNA was used for each reaction. PCR was also performed on RNA and DNase-treated RNA as controls. PCR was carried out in a Techne TC-312 Thermocycler (TechneInc, Burlington, NJ, USA). For each reaction the following program was used: an initial denaturation at 95°C for 5 min; 35 cycles each consisting of denaturation at 95°C for 30 s, annealing at 55°C for 30 s, and elongation at 72°C for 60 s; and a final elongation period at 72°C for 5 min.

qRT-PCR was used to evaluate excision of the SpyCI from the host chromosome and to evaluate transcription of MMR genes, *mutS* and *mutL*. Primers (Table 2) were designed using the Primer3 program^1^ and synthesized by Integrated DNA Technologies (Coralville, IA, USA). For each real-time PCR plate evaluated, primers to a 126 base pair (bp) region of the 16S ribosomal subunit housekeeping gene were also run on each sample for normalization of the data. All data was analyzed with respect to the uninduced, overnight sample for each strain being equal to one. For each reaction, 10 ng of cDNA was used for qRT-PCR analysis of *mutS*, *mutL*, and 16S ribosomal subunit expression. Water blanks were run as negative controls, and all samples and controls were run in triplicate. qRT-PCR was carried out on a BioRad iCycler equipped with the real-time optical fluorescent detection system using SYBR Green PCR Master Mix (BioRad Laboratories, Hercules, CA, USA). The following program was employed for all qRT-PCR: an initial denaturation (95°C, 3 min); followed by 35 cycles of denaturation (95°C, 30 s), annealing (55°C, 30 s), and elongation (72°C, 30 s). Following the last cycle, a melt-curve analysis was performed to verify that a single product was produced for each primer pair. The resulting data was analyzed using Excel and plotted using Prism4.

**Table 2:** **Plasmids and PCR primers**.

Plasmid	Description		Ref.
pMV158GFP	Mobilizable, Gram-positive vector encoding *gfp*		Nieto and Espinosa (2003)
pSMART-LCKan	Low copy, transcription-free cloning vector		Lucigen Corp., Middleton, WI, USA
pGEM-T Easy	PCR cloning vector		Promega Corp., Madison, WI, USA
pDL276	Shuttle vector for low %G + C Gram-positive bacteria		Dunny et al. (1991)
pCAMP17	Phage T12 derived integration vector with a cloning site under the control of the CAMP promoter		Gase et al. (1999)
pWM448	SpyCIM1 *int* clone into the CAMP promoter in pCAMP17		This work
pWM481	SpyCIM5 *int* pseudogene and upstream (primers M5*int*5 and M5*int*4) fused to *gfp* in pSMART-LCKan		This work
pWM483	Insert from pWM481 cloned into pDL276		This work
pWM485	SpyCIM5*int* pseudogene (primers M5*int*1 and M5*int*4) fused to *gfp* in pSMART-LCKan		This work
pWM486	SpyCIM5 *int* pseudogene subregion (primers M5*int*2 and M5*int*4) fused to *gfp* in pSMART-LCKan		This work
pWM487	SpyCIM5 *int* pseudogene subregion (primers M5*int*3 and M5*int*4) fused to *gfp* in pSMART-LCKan		This work
pWM488	Insert from pWM486 cloned into pDL276		This work
pWM489	Insert from pWM485 cloned into pDL276		This work
pWM490	Insert from pWM487 cloned into pDL276		This work

**Sequence**	**Primer**	**Gene target**	**Product size (bp)**

**PRIMERS FOR PCR**			
5′AGCAGGGATTGAACGCTTTA	*attB*-L	*int*-*mutL* intergenic region (*attB*)	655 (strain SF370)
5′TCAATTTCCCCACCAGTAGC	*attB*-R		694 (strain Manfredo)
5′CTTGCCTGCTGAACTCATTG	*attP*-L	SpyCI *att*P	461
5′CACGCTTTTAGACACACTCA	*attP*-R	
5′AAGCGTGAGGTCGTTCAAGT	*mutS*-L	*mutS*	406
5′AGTGGCTGAGTTCTCGCATT	*mutS*-R	
5′GAGGCTTTACCGTCTGTTGC	*mutL*-L	*mutL*	413
5′CCGGAAACTTCAAAATCCAA	*mutL*-R	
5′ATGAGTAAAGGAGAAGAACTTT	*gfp*-L	*gfp* ORF	717
5′TTATTTGTATAGTTCATCCATGC	*gfp*-R	
5′ACAAGCATCACAGGAAGAAC	M5*int*1	SpyCIM5 *int* pseudogene and promoter	Various lengths
5′ATAAGATAACCGCTAGTGATA	M5*int*2	
5′TATTGATCCGTCTGATTTGA	M5*int*3	
5′AATTTTGTTTTTAACACTTTCTAAAC	M5*int*4	
5′CTTACTCCCATTCGGGAACA	M5*int*5	
**PRIMERS FOR qRT-PCR**			
5′ACTGCCAATCCAAGTCCAAT		SpyCIM1 *attP*	96
5′TGAGAGGTGTAAACCTAATGAAACC	
5′TTGAGAAGTGTAAACCTAATGAAACC		SpyCIM5*attP*	112
5′CAATACCCTGTAAAAACTGCCAAT	
5′ACACTCGCTGGCCTTTCTACAACTTCA		*attB*	65
5′CGAGGCCTTCGGCTATTTAT	
5′CTGCGACAGCAACCTTGTAA		*mutS*	108
5′GCAACAAGAATGCGGAAAAT	
5′CCGGAAACTTCAAAATCCAA		*mutL*	111
5′GACATCAGGAACTGGCGATT	
5′AGCGTTGTCCGGATTTATTG		16S rRNA	126
5′CACTCTCCCCTTCTGCACTC	

### Determination of spontaneous mutation rates

A fluctuation test based on the Luria and Delbrück assay (Luria and Delbrück, 1948; Rosche and Foster, 2000) was used to determine the spontaneous mutation rates of 10 *S. pyogenes* strains as previously described (Scott et al., 2008). Strain NZ131 was used as a SpyCI-free control for comparison to the SpyCI containing strains. A single colony of each strain was used to inoculate a THY broth culture and grown overnight at 37°C. The overnight culture then was diluted in THY to less than 1000 CFU/ml and dispensed as 41 1 ml aliquots into culture tubes. Cultures were incubated at 37°C overnight. One tube was used to determine total CFU/ml by diluting in phosphate buffered saline (PBS) and plating dilutions of 10^−4^ and 10^−5^ CFU/ml on THY agar plates in triplicate. The 40 remaining cultures were each mixed with 3 ml of melted soft agar (0.6% agar in water at 45°C) and poured onto THY plates containing 10× the previously determined minimum inhibitory concentration of the gyrase inhibitor ciprofloxacin (2 μg/ml; Scott et al., 2008). For some experiments, 500 μg/ml streptomycin was used for selection of spontaneous antibiotic resistant mutants. Plates were incubated at 37°C for 6–17 days to allow for growth of ciprofloxacin or streptomycin resistant colonies. Plates were examined for colonies each day and were counted on the day when colonies first appeared. Since strain Manfredo never produced antibiotic resistant colonies at this population density, the protocol was modified to use 5 ml replicate cultures for overnight growth that were concentrated by centrifugation and resuspended in 1 ml of THY before plating onto selective medium or diluting for CFU determination. The mutation rate with confidence limits was calculated for each strain using the algorithm of Ma et al. (1992), combined with the technique of maximum likelihood estimation (Stewart, 1994), as implemented by the software package ft (Shaver and Sniegowski, 2003). All experiments were performed at least three times for each strain and the data collected was averaged to determine mutation rate in mutations/generation (μ).

### UV irradiation killing assay

The sensitivity of *S. pyogenes* strains to killing by UV irradiation from a calibrated 254 nm germicidal lamp (120 μW/cm^2^) was done as previously described (Scott et al., 2008). UV irradiation of cultures was carried out in a darkened room to avoid photoreactivation. Assays were conducted a minimum of three times per strain to confirm results.

### Fluorescent primer extension

Overnight cultures of *S. pyogenes* strains started from a single colony were grown in 50 ml THY broth at 37°C for 16 h. Cultures were then diluted 1:20 in 1 l THY broth and incubated at 37°C, and growth was monitored by absorbance. At *A*_600_ = 0.6, samples were collected and quick-frozen using a dry ice-ethanol bath. RNA isolation was performed and treated with DNase as described above. RNA integrity was evaluated at the University of Oklahoma Health Sciences Center Laboratory for Genomics and Bioinformatics using an Agilent 2100 Bioanalyzer (Agilent Technologies, Santa Clara, CA, USA). Fluorescent primer extension was carried out in RNase-free 0.5 ml tubes using 0.2 μM 5′-6-FAM labeled primers. Primers were made to map the 5′ transcriptional start sites of *mutS* and *mutL*; accordingly, primers were designed to anneal so that the 3′ end of the primer would extend toward the 5′ end of the gene. The primers for *mutS*, *mutL*, and SpyCIM5 (ΦMan.5) *int* were designed to anneal to a region of RNA between 180 and 200 bp from the predicted start codon for each gene (Table 2). Primer extension was performed by combining 1 μl of diluted FAM labeled primer with 20 μg RNA and DEPC-treated water to a total volume of 20 μl. Samples were heated at 70°C for 5 min, cooled on ice for 10 min, incubated at 58°C for 20 min, and left at room temperature for 15 min. The following reagents were added to each sample after primers were allowed to anneal: 4 μl dNTPs (10 μM each), 4 μl DTT (0.1 M), 8 μl 5× first strand buffer, 4 μl Superscript II Reverse Transcriptase (all reagents from Invitrogen). cDNA was synthesized by incubating samples for 2 h at 42°C and then precipitated by the addition of 1 μl glycogen (Ambion), 4 μl 3 M sodium acetate, and 100 μl of 100% ethanol. Samples were stored overnight at −20°C and harvested by centrifugation at 4°C at 13,000×*g* for 20 min. Pellets were washed with 70% ethanol and collected by centrifugation as before for 5 min. Pellets were dried to completion on medium heat in a Savant DNA120 concentrator (Thermo Scientific) and submitted to The University of Oklahoma Health Sciences Center Laboratory for Genomics and Bioinformatics for DNA fragmentation analysis on an ABI 3730 capillary sequencer (Applied Biosystems, Foster City, CA, USA). Data was analyzed using Peak Scanner Software (Applied Biosystems).

### Cloning and expression of the SpyCIM1 integrase

The integrase ORF from SpyCIM1 from strain SF370 was amplified by PCR and cloned into the commercial vector pGEM-T Easy (Promega, Madison, WI, USA) to create plasmid pWM447 (Table 2). This PCR reaction introduces a unique *Bam*HI site into the 5′ end of the ORF, allowing gene fusion to the *S. pyogenes* CAMP promoter (Gase et al., 1999). Following digestion of pWM447 with *Bam*HI and *Pst*I to recover the cloned integrase gene, the fragment was ligated to pCAMP17 (Gase et al., 1999) to create plasmid pWM448. This plasmid contains the phage T12 integrase and attachment site as part of the plasmid and will mediate site-specific integration at an unrelated site, the tmRNA gene (McShan et al., 1997). The plasmid pWM448 was introduced in strain Manfredo by electrotransformation (McLaughlin and Ferretti, 1995). For detection of integrase-mediated excision, DNA or RNA was isolated from strains Manfredo, SF370, or Manfredo (pWM448) during early logarithmic growth. PCR specific for *attB* was used to detect excision of the SpyCIM5 in strain Manfredo following introduction of pWM448.

### Construction of SpyCIM5 integrase-reporter fusions

Various lengths of the Manfredo SpyCIM5 *int* pseudogene and upstream region were amplified using PCR and fused to the green fluorescent protein (GFP) to create reporter fusions (primers and plasmids listed in Table 2). The longest PCR product (1458 bp, primers M5*int*5 and M5*int*4) included the entire upstream region, the pseudogene, and the downstream region to the beginning of the *mutL* ORF, and was cloned to create pWM483. Increasingly shorter products were amplified covering only the pseudogene or portions of the pseudogene and the downstream region as shown in Figure 7 (using primers M5*int*1 + M5*int*4, M5*int*2 + M5*int*4, and M5*int*3 + M5*int*4 to create the inserts for plasmids pWM489, pWM488, and pWM490, respectively). The GFP gene (*gfp*) was amplified by PCR from plasmid pMV158GFP (Nieto and Espinosa, 2003). PCR products were synthesized with phosphorylated primers and ligated using standard protocols (Sambrook and Russell, 2001). Following ligation, the desired fusion product was amplified using the downstream *gfp* primer and the desired SpyCIM5 *int* pseudogene primer. The amplified fragments were cloned into the pSMART-LCKan vector (Lucigen, Middleton, WI, USA). The cloned insert was removed by digestion with *Eco*RI and ligated into the *Eco*RI site of shuttle vector pDL276 (Dunny et al., 1991). This plasmid was introduced by electrotransformation into *S. pyogenes* strains Manfredo or NZ131. For fluorescent microscopy, streptococcal strains were grown overnight in BHI supplemented with 5% heated horse serum and 200 μg/ml kanamycin. The cultures were harvested by centrifugation and washed once with PBS. Cells were resuspended in 0.1 volume of PBS, and GFP expression was visualized on a Leica model DM4000B fluorescent microscope using a SPOT digital camera and software package (v. 4.7; Diagnostic Instruments, Inc., Sterling Heights, MI, USA). For mitomycin C treatment, an overnight culture grown in BHI supplemented with 5% heated horse serum and 200 μg/ml kanamycin was diluted 1.20 into fresh media and grown at 37°C until *A*_600_ = ∼0.1. The culture was divided into two aliquots, and mitomycin C (0.2 μg/ml) was added to one aliquot. Incubation was continued for 1 h, the cells were washed and resuspended in PBS as above, and microscopy was performed.

### Bioinformatics

The genome sequences for the SpyCI (annotated as prophages) integrated between *mutS* and *mutL* were extracted from the Genbank annotated bacterial genomes of SF370, MGAS10270, MGAS10750, MGAS6180, MGAS10394, and Manfredo (Table 1). The sequences were aligned and a phylogenetic tree was created using Geneious (Drummond et al., 2012), and the tree was further analyzed by neighbor-network analysis by SplitsTree using the Equal Angle algorithm (Huson, 1998). Promoter predictions were done using neural network promoter prediction software (Reese, 2001) available at the Berkeley Drosophila Genome Project website^2^. Prediction of rho-independent transcriptional terminators in the SpyCI genomes was done using TransTermHP (Kingsford et al., 2007).

## Results

### SpyCI and mutator phenotypes in *S. pyogenes*

In *S. pyogenes* and other Gram-positive bacteria, MMR requires the participation of two proteins, *MutS* and *MutL*, to ensure the fidelity of the genome replication. The loss of MMR function results in a mutator phenotype and can promote homologous or non-homologous recombination in both prokaryotes and eukaryotes (Glickman and Radman, 1980; Rayssiguier et al., 1989; Selva et al., 1997). In group A streptococci, a single polycistronic mRNA is transcribed that includes *mutS* and *mutL* as well as the downstream genes *lmrP*, *ruvA*, and *tag*; the promoter for this operon is immediately upstream of *mutS* (Figure 1). Frequently, a prophage-like chromosomal island (SpyCI) is integrated between *mutS* and *mutL* in *S. pyogenes* genomes, separating *mutL* and the other downstream genes from the promoter upstream of *mutS*. The integration of SpyCI leads to inactivation of the downstream genes, including *mutL*, rendering the bacteria defective for MMR. We recently have shown that in strain SF370, SpyCI integration is dependent on the growth phase of the bacterium (Scott et al., 2008). When cells are rapidly dividing during logarithmic growth, the SpyCI excises from the bacterial chromosome and allows both *mutS* and *mutL* to be transcribed; however, in stationary phase the SpyCI re-integrates and silences the genes downstream of *mutS* (Figure 1). Through this novel system of gene regulation, the SpyCI regulates the streptococcal mutation rate in response to nutrient availability or other environmental signals.

Nine of the 16 sequenced *S. pyogenes* genomes contain a related prophage-like SpyCI between *mutS* and *mutL*, whose presence could result in a complex mutator phenotype. Six of the genome strains were available for this study, and using a modified Luria and Delbrück fluctuation assay (Luria and Delbrück, 1948; Rosche and Foster, 2000) the rate of spontaneous mutation was determined for these *S. pyogenes* genome strains (SF370, Manfredo, MGAS6180, MGAS10270, MGAS10394, and MGAS10750). Genome strain NZ131, lacking a SpyCI, was used as a wild type control as was done previously (Scott et al., 2008). Additionally, clinical M49 strains containing a SpyCI integrated into *mutL* (270fer, 3470-04, and 5474-00) were used as serotype-matched strains to NZ131.

**Figure 2:**
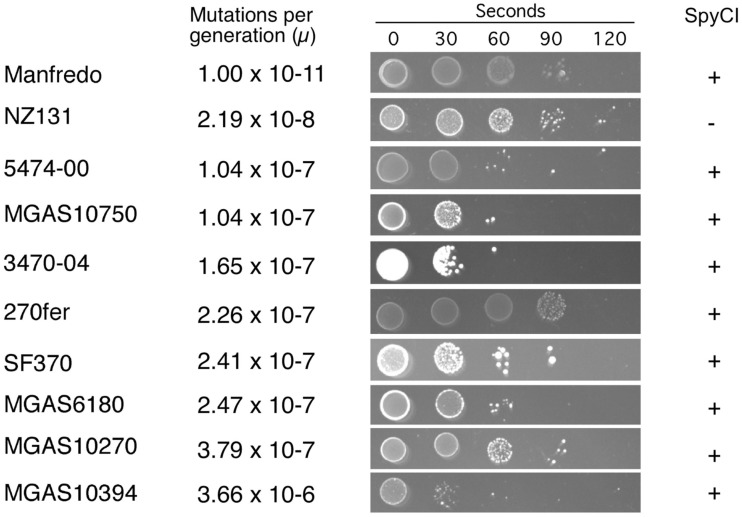
**Mutation rate and UV sensitivity of *S. pyogenes* strains**. The grouping of the *S. pyogenes* strains by mutation rate (μ) is shown as in Table 3. Next to the calculated mutation rate, the sensitivity to UV irradiation is shown for each strain. UV exposure time in seconds is indicated at the top and all strains were plated at the same dilution. All experiments were performed at least three times for each strain.

The mutation rate was estimated to be 2.19 × 10^−8^ mutations/generation (μ) for the non-SpyCI containing strain NZ131 (Figure 2 and Table 3), a value in agreement with our previous report (Scott et al., 2008). Consistent with the hypothesis that a SpyCI located between *mutS* and *mutL* prevents competent MMR, thereby increasing an organism’s mutation rate, the mutation rates for seven of the SpyCI containing strains were determined to be at least one order of magnitude higher than that of NZ131 (MGAS10750, 5474-00, 3470-04, 270fer, SF370, MGAS6180, and MGAS10270). Strain MGAS10394 was found to have a mutation rate of 3.66 × 10^−6^ μ, over 160 times higher than NZ131 (Table 3). The range of observed mutation rates may reflect the variations in the controlling repressor-operator modules in the SpyCI (discussed below) as well as other differences in genetic backgrounds in these strains. Further, the SpyCIM49-containing clinical M49 strains (5474-00, 3470-04, and 270fer) showed an increased mutation rate as compared to NZ131, showing that the phenotype was serotype-independent (Table 3). These studies are highly suggestive, but confirmation of the role of SpyCI in increasing the mutation rate of its host will require the analysis of isogenic strains (manuscript in preparation). The one exception to these findings was that the SpyCIM5 containing strain Manfredo had the lowest mutation rate (1.0 × 10^−11^ μ), suggesting that the presence of the SpyCI did not interfere with MMR in this strain. The mutation rates clustered into three different groups (Table 3): non-mutator with a mutation rate <10^−8^ μ (Manfredo and NZ131); mutator with a mutation rate of ∼10^−7^ μ (MGAS10750, 5474-00, 3470-04, 270fer, SF370, MGAS6180, and MGAS10270); hypermutator with a mutation rate of 10^−6^ μ (MGAS10394).

**Table 3:** **Calculated mutation rates from fluctuation test**.

Strain	Mutation rate in mutations/generation (μ)	Fold difference in mutation rate*	Phenotype
Manfredo	1.00 × 10^−11^	4.6 × 10^−4^	Non-mutator
NZ131	2.19 × 10^−8^	1.0	Non-mutator
MGAS10750	1.04 × 10^−7^	4.7	Mutator
5474-00	1.04 × 10^−7^	4.7	Mutator
3470-04	1.65 × 10^−7^	7.5	Mutator
270fer	2.26 × 10^−7^	10.3	Mutator
SF370	2.41 × 10^−7^	11.0	Mutator
MGAS6180	2.47 × 10^−7^	11.3	Mutator
MGAS10270	3.79 × 10^−7^	17.3	Mutator
MGAS10394	3.66 × 10^−6^	167.1	Hypermutator

*As compared to strain NZ131.

*The mutation rate with confidence limits was calculated as described in the methods. All experiments were performed at least three times for each strain and the data collected was averaged to determine mutation rate in mutations/generation (μ). The mutation rates, as determined by the fluctuation test, clustered into three different groups: non-mutator with a mutation rate <10^−8^ μ (Manfredo and NZ131); mutator with a mutation rate of 10^−7^ μ (MGAS10750, 5474-00, 3470-04, 270fer, SF370, MGAS6180, GAS10270); and hypermutator with a mutation rate of 10^−6^ (MGAS10394)*.

We previously reported that strain SF370 had increased susceptibility to damage from UV irradiation when compared to SpyCI-free strains (Scott et al., 2008). As shown in Figure 1, SpyCI insertion separates *mutL*, *lmrP*, *tag*, and *ruvA* from their promoter upstream of *mutS*. The RuvA protein acts as a sliding collar to prevent the unwinding of Holliday junctions during DNA recombination (Kaplan and O’Donnell, 2006). RuvA then recruits RuvB to the junction to promote branch migration, and the complex subsequently recruits RuvC to resolve the Holliday junction into two discrete DNA duplexes (Parsons et al., 1995; Eggleston et al., 1997; Eggleston and West, 2000). Loss of *ruvA* expression renders a cell sensitive to DNA damage caused by UV irradiation (Iwasaki et al., 1989). As seen in Figure 2, the 10 strains showed a wide range of sensitivities following UV irradiation. In general, the presence of SpyCI in a strain correlated with an increased sensitivity to UV-induced cell death as compared to SpyCI-free strain NZ131. Genome strains SF370, MGAS6180, MGAS10394, and MGAS10750 were at least 100-fold more sensitive to UV killing under the test conditions, with strain MGAS10394 being the most sensitive of all. The SpyCI containing M49 clinical strain 5474-00 and 3470-04 were also sensitive to UV irradiation. Not all of the SpyCI strains were equally sensitive, however. Strain MGAS10270 showed only a slight increase in sensitivity (∼10-fold) while genome strain Manfredo and clinical strain 270fer were at least as resistant to killing as NZ131. Since little is known of the genetic background of strain 270fer, we can only hypothesize that some other mechanism must compensate for the loss of *ruvA*. However, Manfredo again was divergent from the other genome strains and was as UV irradiation resistant as the wild type strains. These unexpected results necessitated further investigation into strain Manfredo.

### Strain Manfredo is defective for site-specific integration

**Figure 3:**
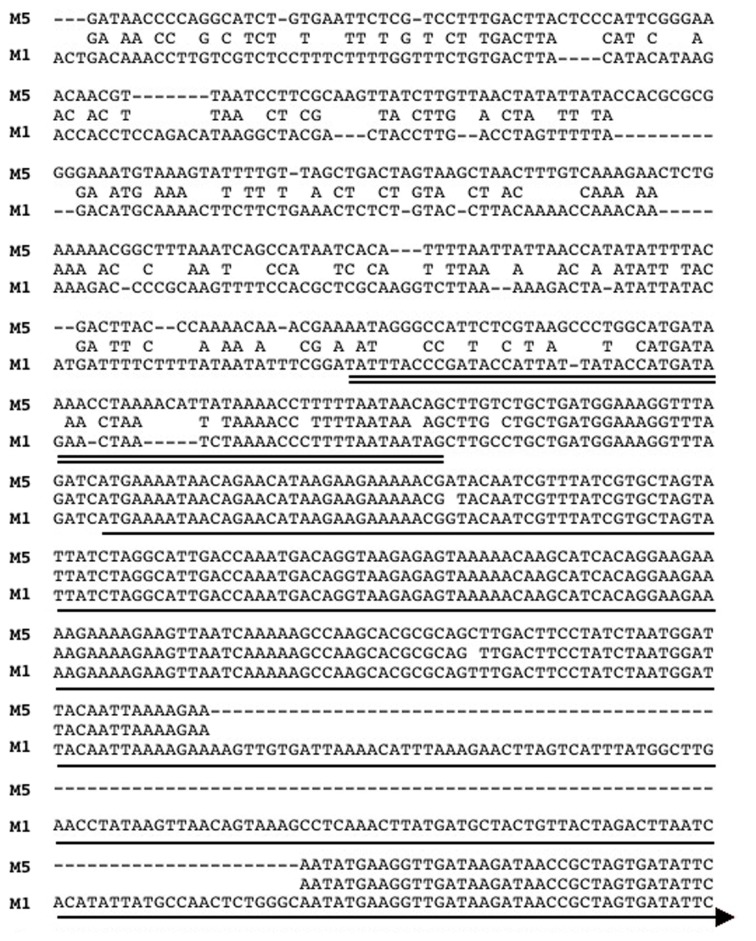
**Alignment of the promoter and 5′ ORF of the integrase genes from SpyCIM1 and SpyCIM5**. The sequence alignment of the promoters and 5′ portion of the *int* coding regions of SpyCIM1 and SpyCIM5 is shown (labeled M1 and M5, respectively). The predicted promoter for SpyCIM1 *int* is double underlined, and the single line indicates the probable beginning of the coding region. The SpyCIM5 promoter has significantly diverged from SpyCIM1 and the other SpyCI-like chromosomal islands. The alignment shows that 128 bp have been deleted from the SpyCIM5 *int*, resulting in not only a loss of genetic information but also the formation of a frameshift. Thus, computational analysis predicts that the Manfredo SpyCIM5 integrase is a non-functional pseudogene.

The only SpyCI containing *S. pyogenes* genome strain that did not have a mutator phenotype or show enhanced sensitivity to UV irradiation was Manfredo. This result was paradoxical since the integrase gene in SpyCIM5 [annotated as ΦMan.5 (Holden et al., 2007)] has a deletion of 128 bp that results in not only loss of coding potential but also introduces a frameshift into this gene (Figure 3); in fact, it is annotated as a pseudogene (Holden et al., 2007). Further, the *int* promoter region in Manfredo is quite divergent from SF370 SpyCIM1 and the other genome strainSpyCI (Figure 3). Since SpyCIM1 integration and excision are crucial to MMR regulation in SF370, the loss of integrase activity in Manfredo could potentially disrupt this growth-dependent process and thus create a permanent mutator phenotype instead of a conditional one as in SF370. However, since such a mutator phenotype was not observed (Figure 2), the potential activity of the integrase gene was investigated.

**Figure 4:**
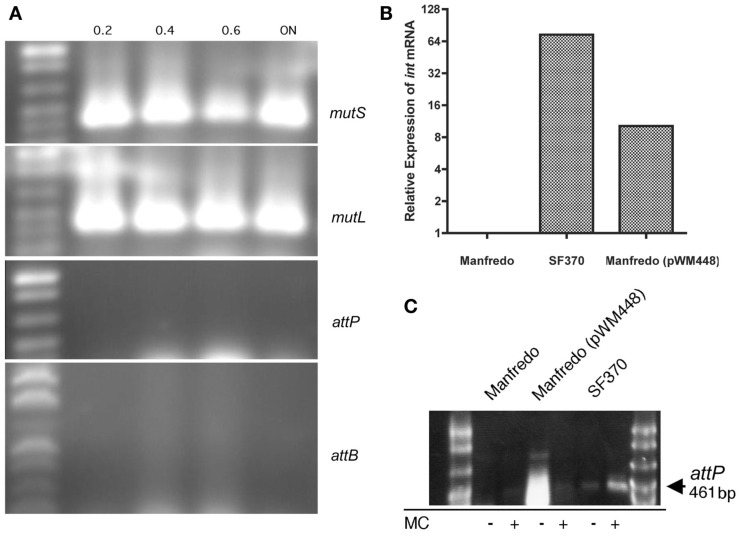
**Expression of *mutL* in Manfredo is independent of SpyCIM5 excision**. **(A)** Expression of both *mutS* and *mutL* in strain Manfredo occurs without SpyCIM5 excision. PCR was used to amplify cDNA from strain Manfredo logarithmically growing cells or after overnight (ON) incubation (the absorbance at *A*_600_ when the cells were harvested is indicated above each sample). Both *mutS* and *mutL* were constitutively expressed while the SpyCIM5-associated sequences of *attP* and *attB* were never detected in Manfredo, indicating that SpyCIM5 excision does not normally occur in response to growth. **(B)** The SpyCIM1 integrase from SF370 was expressed in Manfredo by introduction of plasmid pWM448 containing the SpyCIM1 integrase ORF under control of the strong CAMP promoter. Gene *int* expression is absent from wild type Manfredo, and the target of the qRT-PCR amplification was the deleted region of the integrase ORF not present in Manfredo, confirming that expression was from the cloned SpyCIM1 gene. **(C)** The SpyCIM1 integrase mediates excision of SpyCIM5 in Manfredo. DNA was isolated from early logarithmic grown cells from Manfredo, SF370, or Manfredo (pWM448). PCR specific for *attP*, which is present only when the prophage-like element has excised from the *mutS*-*mutL* junction, produces no product in wild type Manfredo but appears after introduction of pWM448. Mitomycin C (MC), while stimulating the excision of the SpyCIM1 in strain SF370, inhibits the formation of *attP* in Manfredo (pWM448), suggesting inhibition of CAMP promoter activity.

Strain Manfredo was grown to different stages of growth as determined by spectrophotometry, and mRNA was harvested and converted into cDNA. As can be seen in Figure 4A, transcripts for both *mutS* and *mutL* were present throughout growth, and qRT-PCR confirmed that expression was constitutive (not shown). Further, neither mRNA corresponding to the episomal SpyCI *attP* nor the chromosomal *attB* which appear following SpyCI excision could be detected: *attP* is an indication of phage integrase gene activity following excision while *attB* is contained on the wild type polycistronic mRNA containing both *mutS* and *mutL* (see Figure 1). Neither sequence could be detected in strain Manfredo (Figure 4A, *attP* and *attB*) nor could the corresponding encoding regions in the chromosomal DNA be detected by PCR (not shown). The absence of these regions resulting from SpyCI excision is in contrast to strain SF370 where these events are linked to the expression of *mutL* during early logarithmic growth (Scott et al., 2008).

To determine whether the lack of excision was due to the apparent defect in the integrase gene or some other confounding factor, the active integrase gene from SpyCIM1 was cloned into vector pCAMP17 where it was placed under the control of the constitutively expressed CAMP promoter (Gase et al., 1999). This vector also contains the *S. pyogenes* bacteriophage T12 integrase and phage attachment site, mediating integration into the *S. pyogenes* tmRNA gene (McShan et al., 1997). The resulting plasmid, pWM448, would introduce the active SpyCIM1 integrase into the Manfredo chromosome at a distant location and under the control of a promoter that would be unaffected by any SpyCIM5 regulation. Introduction of pWM448 into strain Manfredo led to detectable expression of the SpyCIM1 integrase gene by qRT-PCR (Figure 4B) with resulting appearance of the *attP* sequence resulting from excision of SpyCIM5 (Figure 4C). Thus, the lack of SpyCIM5 excision in strain Manfredo is the result of the deletion in the integrase gene and not from any defects in the attachment sites. No significant difference in the mutation rate or degree of UV sensitivity was observed after introduction of pWM448 (not shown); this was not surprising since Manfredo was essentially wild type for MMR before manipulation. However, mitomycin C treatment, which results in enhanced SpyCIM1 excision in SF370 (Scott et al., 2008), inhibited excision of SpyCIM5, which suggested that other factors such as DNA binding proteins might influence the ability of the integrase to mediate site-specific recombination in Manfredo (Figure 4C).

### Transcription of *mutL* in SF370 and Manfredo

**Figure 5:**
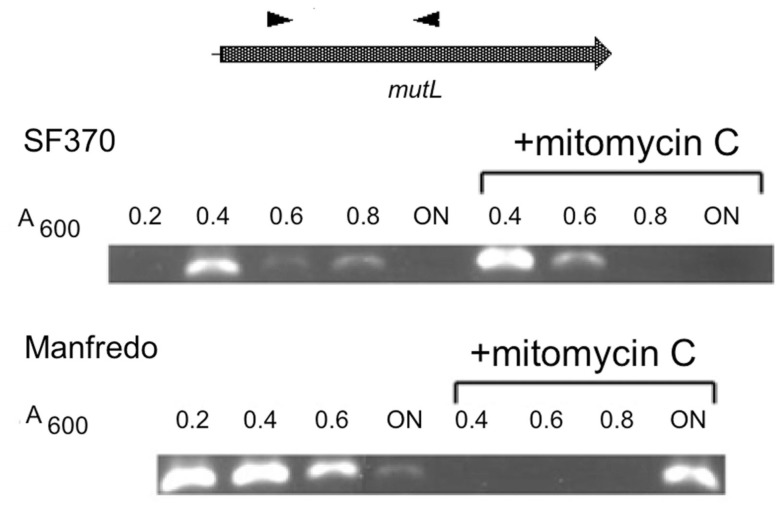
**Manfredo *mutL*mRNA is constitutively expressed under normal growth**. Strains Manfredo and SF370 were grown to increasing cell densities [*A*_600_ = 0.2–0.8 or overnight (ON)]. The mRNA was harvested from the cells, converted into cDNA, and the *mutL* region amplified using primers positioned as shown. For mitomycin C induction, cells were grown to *A*_600_ = 0.2 before mitomycin C was added. In SF370, *mutL* mRNA was transcribed only early in logarithmic phase and then quickly diminished, following the pattern of SpyCIM1 excision and re-integration (Scott et al., 2008). Mitomycin C induction briefly increased the presence of this mRNA. Strain Manfredo, by contrast, expressed this mRNA constitutively throughout logarithmic growth, and small amounts were detectable still after ON incubation. Mitomycin C treatment inhibited expression of the mRNA during logarithmic growth but greatly enhanced its presence after ON incubation.

The lack of detectable SpyCI excision coupled with *mutL* expression and normal MMR phenotype in strain Manfredo suggests that an alternate promoter, possibly of SpyCIM5 origin, controls expression of this gene. Transcriptional analysis by PCR showed *mutL* is transcribed transiently in SF370 and constitutively in Manfredo (Figure 5). Treatment of SF370 with mitomycin C leads to intense expression of this transcript by population-wide SpyCI excision. In neither mitomycin C treated nor untreated SF370 is any mRNA detected once the cells reach deep stationary phase (overnight incubation), confirming the silence of the *mutL* gene in this period of the cell’s life. In Manfredo, transcription of *mutL* is constitutive and the amount of mRNA present gradually decreases as growth slows, but some mRNA is still detectable after overnight incubation (Figure 5). Treatment of Manfredo with mitomycin C inhibits transcription of this mRNA, possibly due to the induction of an antirepressor or other DNA binding protein, but appears to increase mRNA levels in the overnight sample compared to the untreated cells, which may result from the induction of damage repair in these cells. These results, coupled with the lack of detection of SpyCI excision, strongly suggest that *mutL* expression in Manfredo is being promoted via some promoter within the SpyCIM5 that includes at least part of the integrase gene and that regulation of this transcript is fundamentally different from that seen in SF370 SpyCIM1.

### Transcription of *mutL* by a promoter within the SpyCIM5 *int* pseudogene

To identify the promoter activating *mutL* in Manfredo, primer extension was used to map the start of *mutS* and *mutL* mRNA in strains NZ131, SF370, and Manfredo. In all three strains, *mutS* transcription initiates from essentially the same location, with reverse transcription terminating 147 bp upstream of the primer start site in NZ131 and SF370 and 146 bp upstream of the primer start site in Manfredo (not shown). Thus, transcription is initiating about 30 bases downstream of the putative start codon as previously determined by *in silico* analysis of all three genome annotations, placing the transcriptional start site at nucleotide 1,754,003 in NZ131, 1,790,110 in SF370, and 1,778,534 in Manfredo. Primer extension for *mutL* in NZ131, SF370, and Manfredo did not produce any substantial, significant, or reoccurring peaks (not shown), suggesting that the reverse transcription is not terminating within the 500 bp window analyzed by the ABI 3730 capillary sequencer and consistent with a polycistronic mRNA initiating upstream of *mutS*. The presence of this polycistronic message is expected in NZ131, which has no SpyCI, and in SF370, which has SpyCIM1 capable of excising from the chromosome (Scott et al., 2008). In Manfredo, where SpyCIM5 is unable to excise from the chromosome, these data supports the hypothesis that transcription of *mutL* is driven from a promoter within the *int* pseudogene.

**Figure 6:**
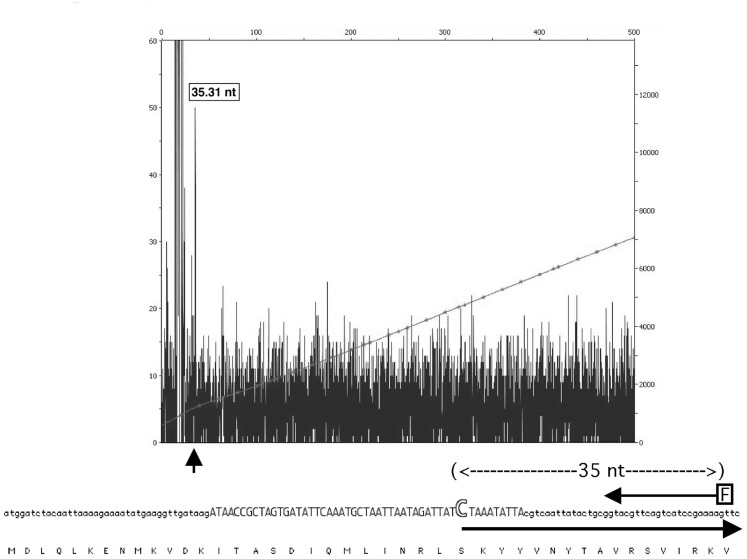
**Mapping of the Manfredo *mutL* transcription to a promoter within the *int* pseudogene**. Primer extension of the SpyCIM5 *int* cDNA revealed the presence of a cryptic promoter within the *int* pseudogene. The fluorescent primer (arrow labeled “F”) extended to an mRNA start position (35 nucleotides upstream) that exactly matched a predicted promoter (Reese, 2001) region (in capitals) and mRNA start (large open “C”) within the pseudogene ORF. The large arrow indicates the initiation and direction of the predicted mRNA. The encoded protein sequence of *int* is shown below for reference. No mRNA initiating from the region upstream of the integrase ORF could be detected. The *X*-axis of the figure corresponds to length of product in basepairs. The left *Y*-axis shows the relative fluorescence units of the sample peaks and right *Y*-axis shows the relative fluorescence units of the GeneScan 600 LIZ size standards. The small vertical arrow indicates the peak corresponding to the product of primer extension.

Primer extension of SpyCIM5*int* cDNA revealed the presence of a promoter within the pseudogene that was completely distinct from the one predicted for *int* in SpyCIM5 or the other genome SpyCI (Figure 6). The pattern of *mutL* gene expression (Figure 5) and the non-mutator phenotype (Figure 2) strongly suggests that this promoter must be constitutively expressed; and this expression pattern is in contrast to SF370 where expression of *mutL* only occurs at a specific point in the growth cycle or following DNA damage. This mapped promoter within SpyCIM5 *int* corresponded to an *in silico* predicted promoter (Figure 6). Transcription from the promoter region upstream of the *int* pseudogene could never be detected by primer extension (not shown).

**Figure 7:**
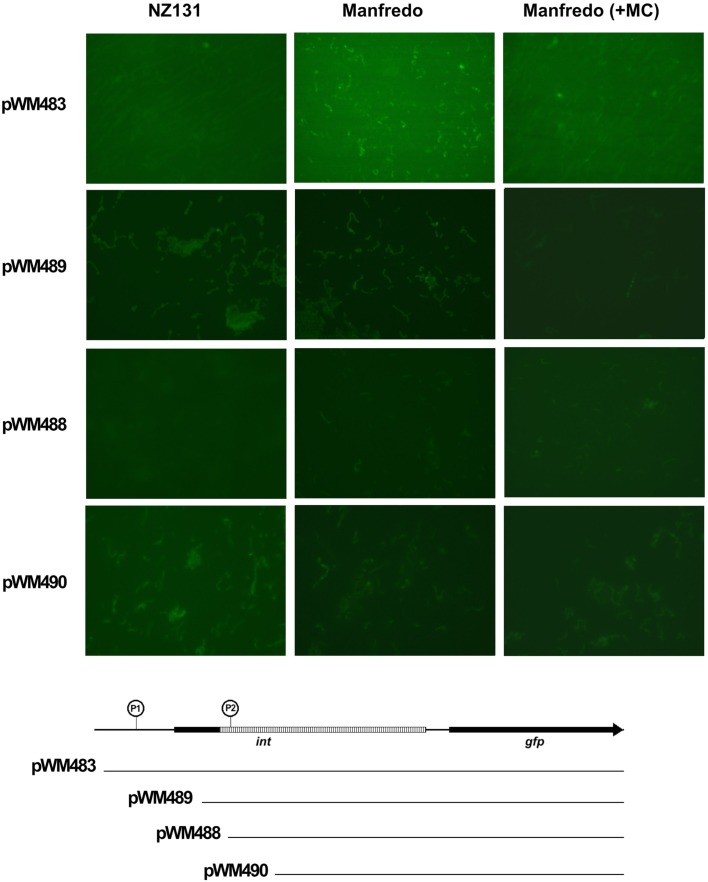
**Transcription from the SpyCIM5 *int* pseudogene is dependent upon host background**. Gene chimeras were made that fused increasing portions of the SpyCIM5 *int* pseudogene and its upstream promoter to the green fluorescent protein gene (*gfp*); the constructs are shown below the micrographs. The downstream region of *int* following the 128 bp deletion is hatched in the figure. An encircled “P” indicates the predicted promoters (Reese, 2001). P1 is immediately upstream of the beginning of the *int* ORF, while P2 is positioned within the pseudogene and maps to the beginning of transcription (Figure 6). The plasmids with these constructs were introduced into strains NZ131 and Manfredo (SpyCIM5+). In *E. coli* JM109, no GFP fluorescence could be detected for any plasmid (not shown).

To test whether the SpyCIM5 *int* pseudogene region was capable of promoting expression of a downstream gene, the region of the Manfredo chromosome that includes the SpyCIM5 *int* pseudogene, its upstream sequence, and downstream through the beginning of the first codon of *mutL* was fused to the GFP gene (*gfp*). This construct was cloned into Gram-positive shuttle vector pDL276 to create plasmid pWM483, which was introduced into strains Manfredo and SpyCI-free strain NZ131. As shown in Figure 7, GFP was expressed in Manfredo but not in NZ131, and the expression varied by cell in Manfredo. Treatment of Manfredo (pWM483) with mitomycin C resulted in the inhibition of green fluorescence, mirroring the inhibition of *mutL* mRNA shown in Figure 5. These results suggest that the activity of this promoter associated with the SpyCIM5 *int* pseudogene is dependent upon the genetic background of the cell, possibly requiring some gene product encoded by the SpyCIM5 itself. Removal of the region upstream of the *int* pseudogene ORF (pWM489) resulted in diminished expression in Manfredo but with some weak expression of GFP in NZ131. Sequentially shortening this region (pWM488 and pWM490) did not restore full expression in Manfredo; mitomycin C treatment had little effect since the cells only expressed low levels without treatment. NZ131 expression continued to be at low levels in these truncated constructs. The results suggest that an enhancer exists in Manfredo that promotes efficient transcription of *mutL* from the *int* pseudogene and that SpyCIM5 may encode this enhancer since SpyCI-free NZ131 only weakly expresses this region. Although the potential enhancer of *mutL* transcription in Manfredo is not identified, treatment with mitomycin C, which induces SOS repair, results in inhibition of GFP expression. Thus, the potential enhancer may be a DNA binding protein that is sensitive to activated RecA cleavages seen in other cells.

## Discussion

The results presented here demonstrate that a SpyCI-associated mutator phenotype is not unique to M1 genome strain SF370. This report examined the effect of SpyCI integration between the MMR genes *mutS* and *mutL* in six genome strains of *S. pyogenes*. We determined the associated mutator phenotypes as measured by spontaneous mutation rate and sensitivity to UV irradiation to better understand the consequences of prophage-like SpyCI integration at this universally conserved site. Further, we presented evidence that in the M5 genome strain, Manfredo, a novel promoter within the SpyCIM5 integrase pseudogene is used to rescue the expression of *mutL* and the downstream genes following CI genome decay. Interestingly, this promoter is inhibited by mitomycin C (and presumably the induction of SOS repair). It is not yet known whether this apparent mechanism of gene regulation is the result of positive selection or merely a circumstantial byproduct of evolution of this compensatory promoter that rescues MMR expression in Manfredo. Collectively, these results provide a snapshot of how SpyCI control of DNA repair in *S. pyogenes* occurs by more than one mechanism, depending upon the integrity of the SpyCI genome integrative functions. These studies provide insight into the co-evolution of prophage-like CIs and their host *S. pyogenes* cells.

Most of the SpyCI containing strains exhibited a mutator phenotype and were at least moderately sensitive to UV irradiation. Strains MGAS10750, 5474-00, 3470-04, SF370, MGAS6180, and MGAS10270 all fell into this category. Since the UV irradiation data is qualitative in nature and may be influenced by other genes in the cell, it is difficult to determine definitively how well the mutation rate correlates with the degree of UV sensitivity. For example, strain 3470-04 appears to be more sensitive to UV light than the rest of the strains in this category, despite having a moderate mutation rate by comparison (Figure 2). It is possible that 3470-04 may have a defect in another DNA repair pathway that is independent of the MMR operon, which contributes to the observed phenotype. Creation of isogenic strains differing only by the presence of SpyCI integrated into *mutL* will help to clarify the specific contribution made by these CIs to the host phenotype.

**Figure 8:**
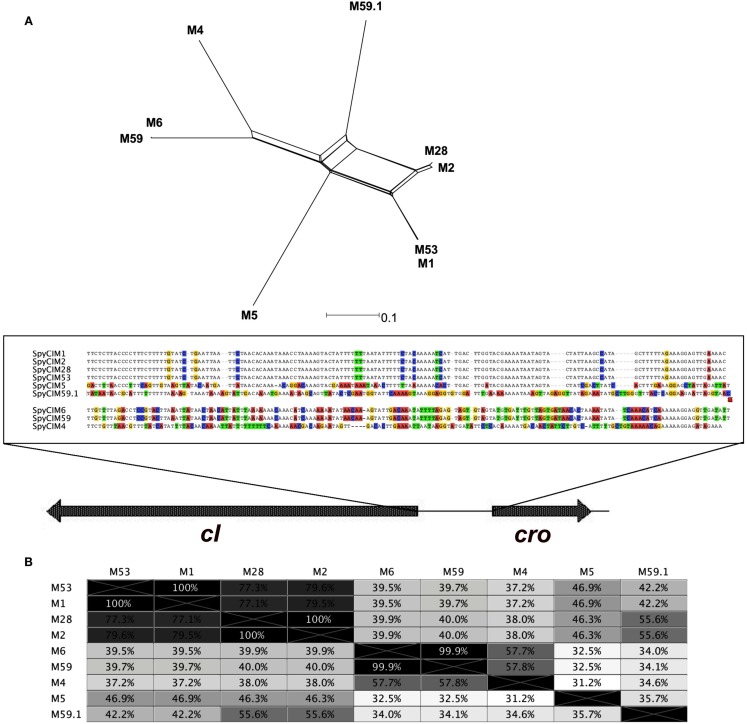
**Phylogenetic analysis of the repressor-operator regions of SpyCI**. **(A)** The chromosomal DNA region of the SpyCI, containing the predicted repressors, operators, and antirepressors, were aligned using Geneious (Drummond et al., 2012), and a phylogenetic tree was constructed from the alignment by neighbor-network analysis (Huson, 1998). Below the tree is the sequence view of the alignment of the operator regions; variants from the consensus are colored by nucleotide. The SpyCI regions analyzed were from strains SF370 (M1), MGAS10270 (M2), MGAS10750 (M4), Manfredo (M5), MGAS10394 (M6), MGAS6180 (M28), Alab49 (M53), MGAS15252 (M59), and MGAS1882 (M59.1). **(B)** Shown is the identity matrix for the DNA alignment of the SpyCI repressor-operator regions.

Of particular interest is strain MGAS10394 with a mutation rate at least a full degree of magnitude higher than all of the other strains. MGAS10394 also has the highest degree of UV sensitivity; only a few colonies survived after any exposure to UV irradiation. The high mutation rate and low tolerance to UV irradiation suggests that the SpyCIM6 may exist more frequently in the integrated state and less as an episome, which would shift the MGAS10394 population toward the mutator phenotype. One of the regions of genetic diversity in the SpyCI is the predicted region controlling lysogeny (i.e., the predicted repressor, antirepressor, and intervening operator sequence). Phylogenetic analysis of this region suggests that this region may be undergoing rapid evolution (Figure 8). A phylogenetic tree built upon the DNA sequence of this lysogeny module reveals that recombination has acted upon this region to create a high level of diversity, and this variation could alter the affinity that the repressor and antirepressor have for the operator DNA sequence as well as their sensitivity to proteases that could lead to cleavage and induction of excision. Further studies are needed to test this hypothesis. The operator sequence between the predicted repressor and antirepressor shows that several groupings exist (Figure 8A). SpyCIM1, SpyCIM2, SpyCIM28, and SpyCIM53 all have nearly identical operator sequences; SpyCIM5 also belongs to this group but has undergone a number of changes at the DNA level. An interesting side effect of tipping the regulation toward integration and the mutator phenotype is that the SpyCI itself would be subject to more frequent mutations, which potentially could lead to inactivation of the CI as seen in SpyCIM5 in Manfredo (assuming that SpyCIM5 was an active CI that mediated a growth-dependent mutator phenotype at some point in its evolutionary past). It is also noteworthy that the recently reported emerging hypervirulent serotype M59 *S. pyogenes* strain associated with invasive disease was remarkable for its lack of typical toxin-associated prophages (Fittipaldi et al., 2012). However, genome comparison of this emerging strain (MGAS15252) to a historic strain (MGAS1882) showed that while both harbor SpyCI integrated into *mutL*, these elements have different lysogeny modules (Figure 8, identified as M59 and M59.1, respectively). The lysogeny module in hypervirulent strain MGAS15252 is now essentially the same module from the hypermutator M6 strain MGAS10394 (Figure 8). MGAS15252 was reported to produce antibiotic resistant derivatives (Fittipaldi et al., 2012), but no estimation of the mutation rate or other details were reported so it is not known whether this strain was a hypermutator. Given that many striking phenotypic changes are associated with MGAS15252 such as enhanced transmission by skin contact, significantly impaired ability to grow in saliva, and a tendency not to colonize the oropharynx (Fittipaldi et al., 2012), the contribution of a SpyCI controlled mutator phenotype to this strain’s emergence should be investigated.

Genome sequencing has established that prophages comprise the majority of genetic diversity between serotype-matched *S. pyogenes* strains (Ferretti et al., 2001; Beres et al., 2002; Smoot et al., 2002). It can be inferred that most of the phenotypic variations between strains of *S. pyogenes* are often due to differential prophage acquisition and gene expression. The SpyCI of *S. pyogenes* are a unique subset of phage-like CIs in various stages of genome evolution or decay. While similar in gene configuration, no two SpyCI are identical. This work suggests that the degree to which each SpyCI responds to or has been silenced by its host results in various phenotypes and gene expression patterns. While the majority of the strains exhibit UV sensitivity and increased rates of mutation as a result of SpyCI integration, there were notable exceptions to this generality: Manfredo, 270fer, and MGAS10394 demonstrated either a wild type phenotype, a mixed mutator phenotype, or a hypermutator phenotype, respectively. These exceptions may represent cases of newly acquired CI that have yet to undergo rounds of selective pressure as well as severely decayed phages that have been optimally streamlined to accommodate a more permanent residence in the host chromosome. The evolution, dissemination, and host range of SpyCI will be important topics for future studies to help clarify the impact that they have on streptococcal survival and virulence.

## Conflict of Interest Statement

The authors declare that the research was conducted in the absence of any commercial or financial relationships that could be construed as a potential conflict of interest.

## Footnotes

^1^http://frodo.wi.mit.edu

^2^http://www.fruitfly.org/seq_tools/promoter.html
